# Mining for viral fragments in methylation enriched sequencing data

**DOI:** 10.3389/fgene.2015.00016

**Published:** 2015-02-04

**Authors:** Klaas Mensaert, Wim Van Criekinge, Olivier Thas, Ed Schuuring, Renske D.M. Steenbergen, G. Bea A. Wisman, Tim De Meyer

**Affiliations:** ^1^Department of Mathematical Modeling, Statistics and Bioinformatics, Ghent UniversityGhent, Belgium; ^2^Department of Pathology, VU University Medical CenterAmsterdam, Netherlands; ^3^Department of Pathology, Cancer Research Center Groningen, University of Groningen, University Medical Center GroningenGroningen, Netherlands; ^4^Department of Gynecologic Oncology, Cancer Research Center Groningen, University of Groningen, University Medical Center GroningenGroningen, Netherlands

**Keywords:** viruses, epigenomics, DNA-methylation, next generation sequencing, bioinformatics, cervical cancer, human papillomavirus, MBD-seq

## Abstract

Most next generation sequencing experiments generate more data than is usable for the experimental set up. For example, methyl-CpG binding domain (MBD) affinity purification based sequencing is often used for DNA-methylation profiling, but up to 30% of the sequenced fragments cannot be mapped uniquely to the reference genome. Here we present and evaluate a methodology for the identification of viruses in these otherwise unused paired-end MBD-seq data. Viral detection is accomplished by mapping non-reference alignable reads to a comprehensive set of viral genomes. As viruses play an important role in epigenetics and cancer development, 92 (pre)malignant and benign samples, originating from two different collections of cervical samples and related cell lines, were used in this study. These samples include primary carcinomas (*n* = 22), low- and high-grade cervical intraepithelial neoplasia (CIN1 and CIN2/3 - *n* = 2/*n* = 30) and normal tissue (*n* = 20), as well as control samples (*n* = 17). Viruses that were detected include phages, adenoviruses, herpesviridae and HPV. HPV, which causes virtually all cervical cancers, was identified in 95% of the carcinomas, 100% of the CIN2/3 samples, both CIN1 samples and in 55% of the normal samples. Comparing the amount of mapped fragments on HPV for each HPV-infected sample yielded a significant difference between normal samples and carcinomas or CIN2/3 samples (adjusted *p*-values resp. <10^−5^, <10^−5^), reflecting different viral loads and/or methylation degrees in non-normal samples. Fragments originating from different HPV types could be distinguished and were independently validated by PCR-based assays in 71% of the detections. In conclusion, although limited by the a priori knowledge of viral reference genome sequences, the proposed methodology can provide a first confined but substantial insight into the presence, concentration and types of methylated viral sequences in MBD-seq data at low additional cost.

## 1. Introduction

The advent of next generation sequencing (NGS) has initiated a revolution in molecular biology. Due to massively parallel sequencing, new insights could be revealed in genetics, transcriptomics and more recently epigenomics. However, the processing of the sheer amount of data produced by these methods proved to be a challenge. Identification of nucleotide sequences is often the first step in many NGS analyses, yet a substantial fraction cannot be properly identified. These unidentified fragments might arise from low-complexity regions (e.g., repeats), bacteria, viruses, other organisms or artificial noise (e.g., adaptor dimers, Head et al., 2014).

Previous studies have identified viruses by screening reads of RNA-seq from human samples. With this approach, the occurrence of EBV and CMV could be demonstrated in colorectal cancer and a landscape of viruses could be identified in a range of cancers (Khoury et al., 2013; Salyakina and Tsinoremas, 2013). In this project, we interrogated fragments from methyl-CpG binding domain enrichment based sequencing (MBD-seq) for a putative viral origin, thereby evaluating whether a similar approach could also be successful for DNA methylation studies.

MBD-seq is a methodology for the detection of CpG-methylation, an epigenetic modification that is essential for cellular differentiation and in processes such as genomic imprinting, X-chromosome inactivation and silencing of transposable elements (Jones, 2012). This method is based on the enrichment of CpG methylated fragments using methyl binding domains followed by massive parallel sequencing. By mapping these fragments to a reference genome, the putatively methylated locus can be determined. Though affected by several biases, the amount of mapped fragments to a locus can be considered as a proxy for the methylation degree of that locus. MBD-seq has been shown to be sufficiently sensitive, specific and cost effective for genome-wide studies (Serre et al., 2010; Aberg et al., 2012).

Viruses play an important role in public health. Aside from causing infectious disease, some are known to be clear risk factors for the development of cancer. Currently known oncoviruses include human papilloma virus (HPV), Epstein-Barr virus (EBV), Kaposi's sarcoma associated herpesvirus (KSHV), Human cytomegalovirus (CMV) and Merkel cell polyomavirus (MCP). It is estimated that viruses have a causal role in about 16% of all human cancers (Schiller and Lowy, 2010; de Martel et al., 2012). Therefore, prevention and vaccination for these viral infections could prevent the occurrence of the cancers they cause. Viral DNA detection has been previously achieved by a range of other methods (Bexfield and Kellam, 2011). State-of-the art methods are particularly sequencing based, for example combined with enrichment techniques or ultra deep sequencing (Allander et al., 2001; John et al., 2011; Lysholm et al., 2012). Enrichment based methods are however dependent on viral particles, which restrains them from detecting integrated viruses. Deep sequencing on the other hand gives an unbiased representation, but severely reduces the efficiency (Willner and Hugenholtz, 2013). With the advent of sequencing based viral research, also the need for specific bioinformatics tools became urgent (Fancello et al., 2012).

CpG methylation is known to play various roles in the life cycle of viruses and their oncogenicity (Hoelzer et al., 2008; Poreba et al., 2011). For example, papillomaviruses are generally hypomethylated when being actively replicated, but are heavily methylated while inserted into the host genome (Hoelzer et al., 2008). HPV might be mediating the methylation of its own genome, as HPV16's viral protein E7 is found to bind and stimulate the activity of DNA methyltransferase 1 (Dnmt1) (Burgers et al., 2007). Also, viral DNA hypermethylation of HPV is more prominent in carcinomas than in asymptomatic infections or dysplasia (Fernandez et al., 2009; Marongiu et al., 2014). In EBV, hypermethylation helps to hide its presence by inhibiting expression of viral latency proteins that could be recognized by cytotoxic T-cells (Paulson and Speck, 1999). Even the latency stage and the tumor type are associated with different methylation patterns of the EBV genome (zur Hausen, 2006). Adenoviruses have also been proven to be *de novo* methylated by insertion, but never in a free DNA stage (Doerfler, 2009). As several tumor-promoting and potentially methylable viruses remain to be identified, we aim at identifying viruses in the typically ignored non-reference aligned sequence reads of MBD-seq experiments.

Here, we demonstrate the usability and relevance of this approach on a collection of cervical samples, including cervical cancer and cervical intraepithelial neoplasia (CIN), which are putative cervical cancer precursors (Steenbergen et al., 2014). Cervical cancer is the third most occurring cancer among women worldwide and estimated prevalences of HPV in cervical cancer range above 99%, strongly supporting the causal role of HPV in cancer development (Walboomers et al., 1999; Ferlay et al., 2010). Cervical tissue is known to be frequently infected by HPV (Clifford et al., 2005) and HPV is often methylated (Hoelzer et al., 2008). Therefore, cervical samples make an ideal test set for the detection of methylated viruses, HPV in particular.

## 2. Materials and methods

### 2.1. Samples and MBD-seq

Of the 92 samples, 39 samples originated from the VU University Medical Center (VUmc) in Amsterdam, further referred to as Set 1. Of this set, 10 samples were obtained from carcinoma, 12 are high-grade cervical intraepithelial neoplasia (CIN2/3), 3 are low-grade cervical intraepithelial neoplasia (CIN1) and 15 originate from cell cultures (See Table 1). These included 2 isolates of primary human foreskin keratinocytes (labeled EK), 10 DNA isolates of keratinocytes transfected with full length HPV16 and HPV18 DNA and the plasmid pcDNAIneo (Invitrogen) (different passages of cell lines FK16A, FK16B, FK18A, FK18B; Steenbergen et al., 1996), 2 DNA isolates of keratinocytes transduced with HPV16E6E7 cloned in the retroviral vector LZRS-MS-IERS-NEO/pBr (Kim et al., 2006; Steenbergen et al., 2013) and the cervical cancer cell line SiHa. In addition, 52 samples were collected from patients visiting the Department Gynecologic Oncology of the University Medical Center Groningen (UMCG), further referred to as Set 2. Of these samples, 12 samples are from carcinomas, 18 from High-grade cervical intraepithelial neoplasia (CIN2/3), 2 from leukocytes and 20 from normal cervical tissue. The two leukocyte samples were pooled samples from each 2 persons. This study has been approved by the ethical committees of UMCG and VUmc, adhering to the declaration of Helsinki.

**Table 1:** **Overview of the histological sample groups and their origin**.

	**Cell culture**	**Carcinoma**	**CIN2/3**	**CIN1**	**Normal**	**Leukocyte**	**Total**
Set 1	15	10	12	2	0	0	39
Set 2	0	12	18	0	20	2	52
Total	15	22	30	2	20	2	91

To obtain the DNA methylation profiles, the MethylCap kit from Diagenode was combined with Illumina Genome Analyzer IIx paired-end sequencing as described in (De Meyer et al., 2013) except for using 500 ng of input DNA instead of 200 ng. Due to data corruption in a compressed format, data for one CIN1 (complete) and one normal (partially) sample were unavailable for further processing. Therefore, only 2 CIN datasets were available, resulting in a total of 91 samples for analysis. Bowtie 1.0.0 was used to subsequently map the obtained paired-end reads (51 bp) from fastq-files to the human reference genome of NCBI v37 (Langmead et al., 2009). A maximum insert size was set at 400 bp and up to 3 mismatches were allowed in the seed sequence to avoid too stringent mapping. For other parameters, the default settings were used. DNA molecules for which the paired-end reads could not be mapped to the reference genome will be further referred to as “non-canonical” fragments, whereas “canonical” fragments will be used to refer to fragments that could be aligned to the reference genome. The non-canonical fragments can be obtained from our website (http://www.biobix.be/viralmbd/).

### 2.2. Virus detection

We aimed to identify fragments of viral origin. This was achieved by searching for sequence similarity between the non-canonical reads and a set of viral reference genomes. For this purpose, we used FR-HIT (Niu et al., 2011). All viral genomes from NCBI and EMBL-EBI were used for the construction of a set of viral reference genomes (http://www.ebi.ac.uk/genomes/virus.html & http://www.ncbi.nlm.nih.gov/genomes/GenomesGroup.cgi) (Wheeler et al., 2006; Leinonen et al., 2011). For reference genomes with a sequence similarity of over 95% (cut-off), the shortest genomes were removed with CD-HIT-EST (Fu et al., 2012). This prevents a bias for those fragments for which there are more similar reference genomes. Mapping of paired reads on different, but very similar reference genomes are not being withheld and would therefore otherwise create false negatives. In order to diminish false positive identifications, several precautions were taken. First, reads featured by low complexity (dust-score >4) were filtered out with prinseq-lite (Schmieder and Edwards, 2011). Second, FR-hit was forced to utilize the complete reads by using the “global mapping” strategy and only the best hits with an *e*-value smaller than 10^−5^ were used. Finally, only if both best hits from each paired-end originated from the same virus, viral identification was affirmed. Duplicated fragments, which have the same start for their first read and the same end position for their second read, were removed. By default, FR-hit masks reference sequences for low complexity regions, however since such a filtering is performed on the reads, this function was disabled. The end result of this approach is a dataset of virus (*v*) specific counts (*N*_*vs*_) for each sample (*s*). Whenever we observed an *N*_*vs*_ > 0, we reported the virus to be present for that sample. Scripts for the execution of the pipeline can be found here: https://github.com/klamens/viral-pipeline.

### 2.3. Statistical tests

Testing for association between histological origin (carcinoma, CIN2/3, CIN1, normal) and the presence of HPV in a sample was performed by Pearson's Chi-squared test with 2000 simulated permutations. Association of high-risk HPV type occurrence and histological groups was tested as well. The most abundant HPV type per sample was used for the assessment of high/low risk HPV type occurrence. When abundances of the most and second most abundant type were equal and their risk was different, the sample was rejected for testing. For a comparison of the fraction of viral fragments, *N*_*vs*_-values were normalized relative to the total fraction of sequenced fragments. These normalized fractions are denoted as *R*_*vs*_. The fractions of counts mapped to specific viruses were compared and tested for with the Kruskal-Wallis Test between the different histological groups. These groups included samples from carcinoma, CIN2/3, CIN1, normal and only for HERV-K113 also cell cultures and leukocytes. *Post-hoc* analyses were performed with the Mann-Whitney-Wilcoxon Test and *p*-values were adjusted for multiple testing by Bonferroni correction (Hochberg, 1988). For both the Kruskal-Wallis Test and the Mann-Whitney-Wilcoxon Test, a location shift assumption was made, resulting in testing for a difference between the medians of *R*_*vs*_. Statistical analyses and graphical plot creations were performed within the statistical environment R (Wickham, 2009; R Core Team, 2012).

### 2.4. HPV type verification

Samples of Set 1 were assessed for HPV (type) presence using the GP5+/6+ PCR followed by enzyme immunoassay (EIA) read-out system using a probe cocktail of 14 high-risk HPV types (HPV16, 18, 31, 33, 35, 39, 45, 51, 52, 56, 58, 59, 66, and 68) (Jacobs et al., 1997). Reverse line blot was used to genotype all EIA-positive samples (van den Brule et al., 2002) using probes for HPV-types 6, 11, 16, 18, 26, 30, 31, 33, 34, 35, 39, 40, 43, 45, 51, 52, 53, 54, 55, 56, 57, 58, 59, 61, 66, 67, 68, 69, 70, 71, 73, 81, and 82. Samples of Set 2 were tested for presence of high-risk HPV-DNA with both the HPV GP5+/6+ general primers, and HPV16- and HPV18-specific primers (Wisman et al., 2006) as performed routinely in the ISO-15189 accredited laboratory. In all tests a serial dilution of DNA extracted from CaSki (ATCC; CRL1550; 500 integrated HPV16 copies), HeLa (ATCC; CCL2; 20–50 integrated HPV 18 copies), SiHa (ATCC; HTB35; 1–2 integrated HPV16 copies), CC10B (HPV45-positive cell line) and CC11 (HPV67 positive cell line), and HPV-negative cell lines were included as control for the analytical specificity and sensitivity of each hrHPV-PCR (Tjon Pian Gi et al., 2014). To assess the MBD-seq based HPV type identification, concordances for samples with and without the specific HPV types were calculated.

## 3. Results

### 3.1. Non-canonical fragments

**Figure 1:**
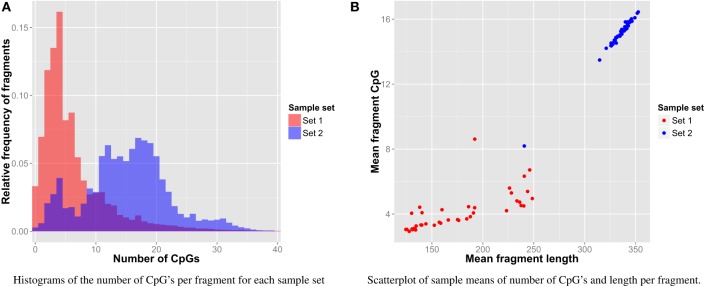
**Fragment CpG content**. **(A)** Histogram of CpG content per sample set. **(B)** Relation of CpG content and length per sample.

On average, 29% (*SD* = 9%) of all fragments in each sample could not be aligned to the human reference genome. Of these reads, only 0.31% (*SD* = 0.17%) could be mapped to the viral reference genomes. In total, we tried to map reads of 4.3×10^8^ non-canonical fragments to 6433 different viral genomes, obtained after removal of very similar genomes (see Materials and Methods). More details about the mapping statistics can be found in the Supplementary Material. As MBD-seq enriches for methylated CpGs, a high-quality dataset should include only a limited amount of fragments without any CpG, and most fragments should have multiple CpGs (De Meyer et al., 2013). This holds for both sample sets (1 and 2) as depicted in Figure 1A. Differences in the number of CpGs per fragment per sample between Sets 1 and 2 can be explained by differences in fragment length (Figure 1B). Overall, this analysis suggests that most identified viruses (see below) are indeed methylated.

### 3.2. Detected viruses

In a first phase, the presence of specific viruses in the different sample sets was assessed (see Table 2). For all samples, fragments similar to the human endoretrovirus K113 (HERV-K113) could be identified. However, as sequence identities of mapped reads with HERV-K113 were sometimes as low as 75%, it is most likely that these fragments originate from other HERV-Ks as well. A significant difference in *R*_*vs*_ for these HERV-K113 similar fragments could be demonstrated between the histological groups (*p < 0.0001*), but *post-hoc* tests revealed only significantly higher HERV-K113-like fractions in the cell cultures compared to normal tissue, CIN2/3 and carcinomas (all *p* ≤ 0.001, data not shown).

**Table 2:** **Sample counts (*N*_*vs*_) of relevant identified viruses**.

	**Cell culture**	**Carcinoma**	**CIN2/3**	**CIN1**	**Normal**	**Leukocytes**	**Total**
HERV-K113	15	22	30	2	20	2	91
phage phiX174	7	20	29	2	20	2	80
Human adenoviruses	5	10	9	2	1	0	27
Merkel cell polypmavirus	0	0	2	0	0	0	2
Epstein-barr virus	0	6	4	0	1	0	11
Human cytomegalovirus	7	0	0	0	0	0	7
Human herpesvirus 1	0	1	0	0	0	0	1
Human herpesvirus 6	0	1	0	0	0	0	1
Human herpesvirus 7	0	0	0	1	0	0	1
Human papillomavirus	14	21	30	2	11	1	79

Also phages were frequently observed in various samples, though in very low abundances in all cell culture samples and CIN1 samples. Enterobacteria phage PhiX174 is the most occurring phage. This isn't surprising, as PhiX174 is being used as a spike-in for quality and calibration control in the Illumina sequencing protocol. Other phages that were observed at lower levels were, among others, phage lambda and phage P1 (data not shown).

Human adenoviruses were discovered in multiple samples. More fragments were observed in samples originating from Set 1 compared to those of Set 2. The most occurring types were human adenovirus C and human adenovirus B. Two CIN2/3 samples contained a single fragment of the Merkel cell polyomavirus. Multiple, particularly carcinoma and CIN2/3, samples were found to contain one to 25 fragments of the Epstein-Barr virus. Human cytomegalovirus was only detected in cell culture samples. However, these fragments most likely originate from the CMV promoter which is included in the pcDNAI neo plasmid. Human herpes virus 1, 6, and 7 were also identified, each in just a single sample.

HPV was detected in all but one sample in the carcinoma group and the cell culture group. It was discovered in all samples originating from the CIN2/3 group and in 11 of the 20 normal samples. Also in both samples of CIN1 and in one of the two leukocyte samples HPV was detected. Association between the presence of HPV and cervical groups (excluding cell culture samples and leukocytes) was assessed for by Pearson's Chi-squared test with simulated (*p* < 0.001).

**Figure 2:**
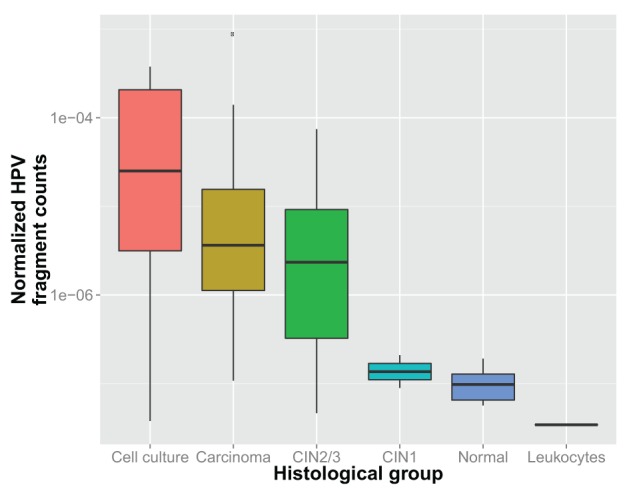
**Normalized HPV fragment counts (*R*_*vs*_) within each sample for which HPV was found, per histological group**.

Next to assessing the (differential) presence of specific viruses, also a quantitative analysis can be performed. To illustrate the feasibility, HPV *R*_*vs*_ in HPV-positive samples were compared between the carcinoma, CIN2/3, CIN1 and normal groups (see also Figure 2). A significant difference between these groups was demonstrated (*p* = 0.0001). *Post-hoc* analyses reveal significant differences between the normal group and cell carcinoma and CIN2/3 samples (see Table 3). It should be noted that the absence of significance for the comparisons with CIN1 may be explained by a lack of power (*n* = 2).

**Table 3:** **Comparison of HPV fragment counts between cervical histological groups**.

	**Carcinoma**	**CIN2/3**	**CIN1**
CIN2/3	1		
CIN1	0.4	1	
Normal	<10^−5^	<10^−5^	0.8

*Values are p-values obtained by post-hoc Mann-Whitney-Wilcoxon test and adjusted by Bonferroni correction*.

**Figure 3:**
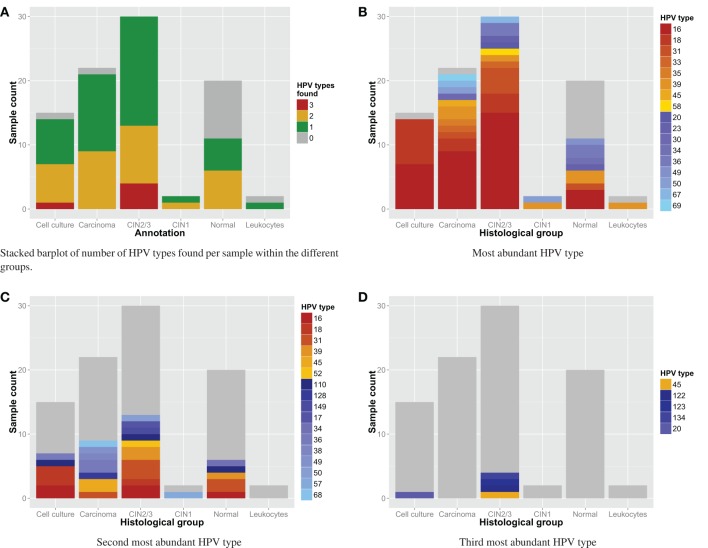
**(A)** detection of HPV types: number of HPV types found per sample. **(B–D)** Stacked barplot of HPV types found per sample with n-th most fragments within the different groups. Red to gold and blue colored types correspond with respectively high and low risk HPV types.

Often multiple HPV types were detected per sample as can be observed in Figure 3A and Table 4. In Figures 3B–D one can see which HPV types were detected in each histological group. The most detected HPV type in primary cervical samples was HPV16 (*N* = 30), followed by HPV31 (*N* = 12), HPV39 (*N* = 9), HPV18 (*N* = 6), and HPV36 (*N* = 6). Other HPV types could not be observed in more than 4 different samples (see Figure 3). We observed a higher relative occurrence of high-risk HPV types in carcinoma and CIN2/3 samples with HPV compared to normal samples, but the association was not significant.

**Table 4:** **Overview of the number of identified HPV types in the different sample groups**.

	**Cell culture**	**Carcinoma**	**CIN2/3**	**CIN1**	**Normal**	**Leukocyte**	**Total**
No HPV	1	1	0	0	9	1	12
1 HPV type	7	12	17	1	5	1	43
2 HPV types	6	9	9	1	6	0	31
3 HPV types	1	0	4	0	0	0	5
Total	15	22	30	2	20	2	91

**Figure 4:**
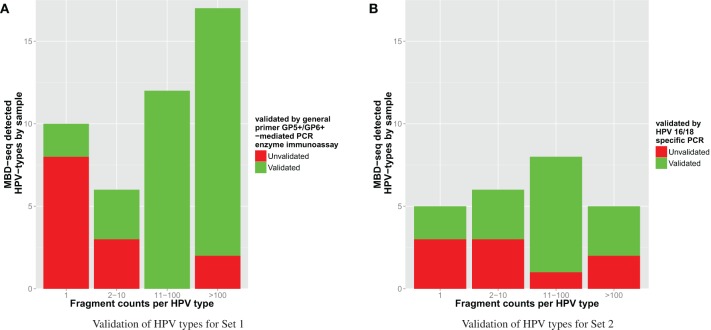
**HPV type validation**.

Though the HPV type analysis yielded relevant results, the overall accuracy of this approach should be evaluated as well. Therefore, verification of the HPV types was performed using independent methods (see Materials and Methods), which we consider here as gold standard. The independent validation of the HPV types yielded a positive verification in 71% of the detections. For HPV types indicated to be present by these methods, results were 66% concordant with the MBD-seq approach. Vice versa, verified absence of viruses was 98% concordant with the proposed methodology. The more fragments that were detected for an identified HPV type, the more likely it was to be validated as can be seen in Table 5. As the verification methodology differed between Sets 1 and 2, results per sample collection can be observed in Figure 4.

**Table 5:** **Overview of the number of validated HPV types according to the amount of detected fragments**.

	**1**	**2–10**	**11–100**	**>100**	**Total**
Unvalidated	11	6	1	4	22
Validated	4	6	19	18	47
Total	15	12	20	22	69

## 4. Discussion

In this study, we demonstrated that the non-canonical fraction of MBD-seq fragments can be used to identify viruses. Considering the increasing importance of sequencing methods, this strategy can provide key evidence regarding the involvement of specific viruses in pathologies at minimal additional cost. Given the roles of DNA methylation in virus biology, the outlined pipeline is capable to generate valuable hypotheses from otherwise unused data. As the outlined application has also several disadvantages (see below), the generated hypotheses should of course be additionally validated by state-of-the art methods. The observed CpG content in many cervical samples, in comparison with De Meyer et al. (2013), suggests that most viral mapped fragments are methylated. It should be noted that unmethylated viral fragments picked up as “noise” may also be relevant, but that the sensitivity for these fragments will most likely be too low to link it to the specific pathology under study.

Recently, some studies already achieved viral identification in RNA-seq experiments by comparable methods (Chen et al., 2013; Salyakina and Tsinoremas, 2013). These studies could find substantial presence of oncoviruses by their transcripts. However, integrated viruses may be temporarily transcriptionally silent, often by DNA methylation, making the proposed methodology a good complement to RNA-seq for viral identification as transcriptionally silenced viruses will also be detected. Moreover, it is capable of revealing epigenetic information about the clinical virus biology. Our method is generic and could be used in combination with other NGS techniques. However, FR-HIT does not account for splicing events which might restrict its applicability to RNA-seq data.

The outlined approach was used on cervical samples of different origin, both histologically and study-wise, and multiple viruses were detected. Not unexpectedly, fragments originating from HERV-K were observed in every sample, which can be considered as a positive control as HERV-K is an endogenous retrovirus (Hohn et al., 2013). Significantly more HERV-K fragments could be observed in the cell culture samples vs. normal tissue, CIN2/3 and carcinomas, which might reflect methylation differences between cell culture and primary samples (Smiraglia, 2001; Varley et al., 2013). This result therefore provides a first indication that also a quantitative comparison of virus count data may yield relevant information. Other expected detections include Phage PhiX174 DNA from Illumina spike-ins and CMV that originated from the pcDNAI neo plasmid in cell culture samples. Indeed, plasmids have been shown to be methylated, which can interfere with specific experiments (Hong et al., 2001).

Interestingly, we detected several oncoviruses in the cervical samples besides HPV. Merkel cell polyomavius, known to cause the Merkel cell sarcoma, was found to be present in two CIN2/3 samples (Feng et al., 2008). Another identified oncovirus is the Epstein-Barr virus. Although not significant, an apparent association between the presence of the Epstein-Barr virus and histological group hints toward its oncogenic role in cervical cancer as has been stated in (Szostek et al., 2009). However, since the counts for Epstein-Barr were low, viral fragments originating from infiltrating lymphocytes is at least an equivalent alternative (Grywalska et al., 2013). Results from this study therefore indicate that additional research should be performed regarding the impact of Epstein-Barr virus and Merkel cell polyomavirus superinfection in CIN2/3 and carcinomas, preferably in far larger groups.

The most prevalent oncovirus however, as expected, was HPV. As the prevalence of HPV in cervix and its causal role in cervical cancer is well documented, the virus detection efficiency of the proposed methodology verifies the capabilities of our method (Clifford et al., 2005; Armstrong, 2010). The role of HPV in cervical cancer was shown by two comparisons. First, there is a significant association of HPV occurrence and histological group. Second, in HPV positive samples we observed a significant increase in total HPV fragments per sample in cell culture, carcinoma or CIN2/3 samples vs. normal samples. The latter observation could be due to more HPV and/or more HPV methylation. More DNA methylation of the HPV genome in carcinomas is in accordance with observations for HPV16 and HPV18 as reported by Fernandez et al. (2009).

However, note that the quantitative evaluation of methylated viruses may also be affected by the global genomic methylation state. Genomic hypermethylation, as often observed in cell lines (Smiraglia, 2001), might suppress viral estimates as their relative abundance in the total methylated fraction may drop. On the other hand, overall hypermethylation may also lead directly to increased viral methylation, and therefore higher sensitivity for MBD-based capturing. A similar reasoning may be relevant for tumor samples, which might feature global hypomethylation (Li et al., 2014). In other words, the overall methylation state will have an important impact, but the exact effect depends on how much viral methylation itself or the detection of methylation is affected by it.

Phages were detected in primary samples from both sample sets and were absent in all cell culture samples. This is not unexpected as the female genital tract is featured by complex microbiological flora and phage genomes have since long been reported to be methylated (Krüger and Bickle, 1983; Martin et al., 2012). The presence of human adenoviruses might be explained by contamination. Both human adenovirus B and C are known to play a role in respiratory diseases, which might explain a possible way of contamination (Jones et al., 2007). The remarkable difference of human adenovirus fragment occurrence between the sample sets reinforces this hypothesis. Observation of HPV in one leukocyte sample might be explained by contamination as well.

Hence, for virus detection with a low fragment count, one should be cautious in concluding viral presence. The high sensitivity of NGS will cause the results regarding presence or not to be easily affected by contamination Yozwiak et al. (2012). For example, HPV39 was detected several times at low fragment count in samples that were run in the same illumina Genome Analyzer lane as one sample with a remarkable high HPV39 fragment count. Also, the high amount of HPV39 positive samples seems to deviate from its relative low prevalence in Europe, this in contrast with the other HPV types (16,18,31) (Clifford et al., 2005). These fragments were most likely categorized in the wrong sample due to carryover associated with common inaccuracies in Illumina multiplex sequencing (Kircher et al., 2012). Improper identifications due to wrong mapping is less likely as viral genomes with high similarity were represented by only one reference genome per group. Furthermore, we checked some of the single HPV hits by blasting them to NCBI nucleotide archive which gave us best hits for the found HPV's. Contamination might therefore partly explain the seemingly high superinfection rate of HPV types. One might therefore opt to only call virus presence upon identification with a minimum fragment count, for example 10 (as also suggested by Yozwiak et al., 2012 and Salyakina and Tsinoremas, 2013). Additionally, the use of double indexing during Illumina multiplex sequencing will remove a major experimental source of carryover contamination (Kircher et al., 2012). For example, HPV detections in samples of Set 1 with more than 10 fragments could all but two be verified. Alternatively, next to contamination, MBD-seq might also be featured by a higher sensitivity due to enrichment for methylation, compared to the methylation naive verification methods. However, it will likely not detect viruses of which no methylated DNA is present.

Another limitation of this best mapping hit based approach is that it enterily depends on existing known viral genomes. In this study, only full genomes of NCBI and ENA were used. However, as the portion of sequenced genomes (6433 in our dataset) is very limited compared to the amount of mammalian viruses estimated at 320.000 (Anthony et al., 2013), it is very likely that many viruses will be missed by this method. Related viruses can be detected by lowering the stringency of sequence similarity. However, this implies an increasing difficulty to distinguish viral types. Distinct viral types will also be harder to distinguish when the set of reference genomes increases as more similar genomes enter. This problem can be solved by clustering and removing similar genomes or by technological advances that increase the length of the sequenced reads. Finally, also horizontal gene transfer or ancestral viral integrations may create false positives. *De novo* assembly of viruses using unmapped fragments largely avoids the dependency on current knowledge and mapping problems, but will require large coverages to obtain sufficient amounts of viral fragments and will be hampered by unmethylated regions of the viral genome.

Generally, we can conclude that this method is effective in detecting fragments of methylated viral DNA. This could be verified by HPV detection in the cervix case study, demonstrating (i) association of HPV presence and histological group (ii) differential quantities of HPV fragments in HPV positive samples between normal samples and carinoma or CIN2/3 samples (iii) type detection with good concordance as verified by independent methods. In other words, if the impact of HPV in cervical cancer would have been unknown, it might have been picked up by the outlined approach, though additional validation would of course have been absolutely necessary. It is therefore clear that the methodology can generate novel knowledge regarding the presence of viruses in disease, and that the inherent disadvantages are by far outweighed by the major benefit of obtaining information regarding the presence of any sequenced virus in otherwise typically discarded data.

## Author contributions

Ed Schuuring, Renske D. M. Steenbergen and G. Bea A. Wisman provided the data regarding the cervical samples and performed the HPV type verifications for these samples. Tim De Meyer and Klaas Mensaert conceived the general idea and approach. Klaas Mensaert and Tim De Meyer have designed the pipeline, analyzed the data and wrote the manuscript. Wim Van Criekinge and Olivier Thas have contributed to the conceptual development and provided critical advice. All authors have reviewed the article and approved the final manuscript.

### Conflict of interest statement

The authors declare that the research was conducted in the absence of any commercial or financial relationships that could be construed as a potential conflict of interest.
