# Supplementary material for: Mining for viral fragments in methylation enriched sequencing data
Source: Front Genet. 2015 Feb 4;6:16. doi: 10.3389/fgene.2015.00016 (PMC4316777; doi:10.3389/fgene.2015.00016)
Supplement: Supplementary file 1 [file DataSheet1.PDF]

ADDENDUM

sample	histological origin	input fragments	uniquely mapped (human)	multiple mapped (human)	unmapped (human)	viral mapped
1	Normal	17159073	54.24%	7.28%	38.47%	0.27%
2	Normal	19593669	52.82%	7.72%	39.46%	0.22%
3	CIN2/3	12161142	54.27%	7.44%	38.29%	0.26%
4	Normal	18704905	55.07%	7.39%	37.53%	0.26%
5	Leukocytes	29202112	55.21%	7.38%	37.42%	0.25%
6	CIN2/3	12470519	56.21%	7.42%	36.37%	0.29%
7	CIN2/3	13820896	56.67%	7.03%	36.31%	0.30%
8	Carcinoma	13044564	55.10%	7.84%	37.06%	0.55%
9	Normal	15711901	55.22%	7.70%	37.08%	0.30%
10	CIN2/3	19958571	55.30%	6.84%	37.85%	0.27%
11	Normal	31019016	55.28%	7.72%	37.00%	0.29%
12	CIN2/3	30021794	56.22%	7.11%	36.67%	0.25%
13	Normal	13483125	67.95%	8.33%	23.73%	0.43%
14	Normal	23302399	67.68%	7.53%	24.79%	0.34%
15	Normal	16355110	67.59%	7.60%	24.81%	0.41%
16	Normal	25281190	68.58%	7.35%	24.06%	0.40%
17	Normal	26808890	68.33%	7.51%	24.16%	0.39%
18	CIN2/3	22762906	67.57%	7.92%	24.51%	0.44%
19	CIN2/3	14889782	69.04%	7.62%	23.34%	0.45%
20	Carcinoma	16427672	68.23%	7.55%	24.22%	0.44%
21	CIN2/3	15588703	66.79%	8.97%	24.24%	0.45%
22	Normal	17577027	65.79%	9.26%	24.95%	0.38%
23	Carcinoma	15640199	68.31%	8.83%	22.85%	0.47%
24	CIN2/3	19639974	66.94%	8.54%	24.51%	0.38%
25	Carcinoma	15015369	63.75%	7.95%	28.30%	0.41%
26	Normal	20504400	66.41%	7.27%	26.32%	0.35%
27	Carcinoma	16870779	65.51%	7.81%	26.68%	0.41%
28	Carcinoma	19868261	66.24%	7.43%	26.34%	0.42%
29	Normal	15256152	64.99%	8.06%	26.96%	0.37%
30	Normal	25280609	66.07%	7.07%	26.87%	0.42%
31	CIN2/3	11498363	66.25%	7.33%	26.43%	0.42%
32	CIN2/3	21535903	66.16%	7.29%	26.55%	0.41%
33	Carcinoma	21191996	65.61%	7.33%	27.06%	0.49%
34	CIN2/3	16036144	66.61%	6.93%	26.46%	0.43%
35	Carcinoma	22494644	66.31%	8.01%	25.67%	0.41%
36	Normal	23447686	66.65%	7.25%	26.09%	0.38%
37	Carcinoma	23164944	60.60%	6.79%	32.61%	0.75%
38	Carcinoma	20482844	59.74%	7.04%	33.22%	0.67%
39	Carcinoma	13859161	58.65%	6.34%	35.01%	0.68%
40	Normal	17641917	58.20%	7.17%	34.63%	0.64%
41	Normal	21298839	66.91%	7.66%	25.43%	0.50%
42	Normal	15515958	65.56%	7.86%	26.58%	0.41%
43	CIN2/3	19980643	66.90%	6.74%	26.35%	0.48%
44	Normal	5238702	67.71%	7.75%	24.54%	0.51%
45	CIN2/3	22794638	66.13%	7.27%	26.61%	0.45%
46	CIN2/3	22877694	64.82%	8.52%	26.67%	0.48%
47	Carcinoma	14477425	69.31%	7.32%	23.37%	0.56%
48	CIN2/3	16300877	66.17%	7.69%	26.14%	0.49%
49	Leukocytes	15436208	67.19%	7.68%	25.13%	0.55%
50	CIN2/3	17133199	67.17%	7.74%	25.08%	0.53%
51	Normal	12676107	66.89%	7.77%	25.34%	0.54%
52	CIN2/3	16454868	67.20%	7.70%	25.10%	0.51%
53	Cell culture	9068647	67.91%	8.13%	23.97%	0.16%
54	Cell culture	8643463	70.28%	7.33%	22.39%	0.20%
55	Cell culture	5165383	66.89%	7.58%	25.53%	0.31%
56	Cell culture	7687200	70.66%	6.81%	22.53%	0.28%
57	Cell culture	7340274	68.16%	7.51%	24.33%	0.37%
58	Cell culture	8343844	66.91%	8.04%	25.06%	0.17%
59	Cell culture	5219303	67.24%	7.99%	24.77%	0.18%
60	Cell culture	6054681	70.97%	6.39%	22.64%	0.34%
61	Cell culture	7674011	68.39%	7.77%	23.84%	0.18%
62	CIN2/3	8318172	64.26%	7.86%	27.88%	0.15%
63	Cell culture	8937760	73.93%	7.08%	18.99%	0.24%
64	Cell culture	9663428	68.13%	8.18%	23.69%	0.14%
65	Cell culture	15502925	63.14%	7.15%	29.71%	0.12%
66	CIN2/3	13826672	58.38%	5.11%	36.51%	0.11%
67	CIN2/3	19491764	57.70%	5.82%	36.47%	0.09%
68	CIN2/3	15353830	51.63%	5.62%	42.75%	0.07%
69	CIN2/3	18961180	49.70%	5.86%	44.44%	0.08%
70	CIN2/3	19188496	66.09%	6.13%	27.78%	0.13%
71	CIN2/3	8853713	60.98%	7.31%	31.71%	0.12%
72	CIN2/3	7366686	65.96%	7.40%	26.64%	0.14%
73	CIN2/3	9744209	63.16%	7.16%	29.68%	0.15%
74	CIN2/3	10239830	58.72%	7.13%	34.15%	0.12%
75	CIN2/3	11727139	59.99%	6.94%	33.08%	0.13%
76	CIN2/3	10161162	57.63%	7.34%	35.03%	0.10%
77	CIN1	11210803	38.38%	6.05%	55.57%	0.06%
78	CIN1	9580411	37.12%	5.97%	56.91%	0.08%
79	Carcinoma	9286556	23.03%	6.81%	70.16%	0.03%
80	Carcinoma	12212800	61.89%	8.30%	29.81%	0.11%
81	Carcinoma	9053466	66.85%	6.70%	26.45%	0.12%
82	Carcinoma	11557226	67.45%	6.72%	25.83%	0.15%
83	Carcinoma	7906767	66.18%	6.99%	26.83%	0.11%
84	Carcinoma	10606384	66.48%	6.47%	27.05%	0.15%
85	Carcinoma	13259982	66.69%	7.19%	26.11%	0.12%
86	Carcinoma	14609723	69.74%	6.85%	23.41%	0.19%
87	Carcinoma	14491374	67.01%	7.23%	25.75%	0.14%
88	Carcinoma	15197698	70.25%	7.05%	22.71%	0.15%
89	Cell culture	44084191	71.02%	6.33%	22.66%	0.90%
90	Cell culture	37526005	71.78%	5.56%	22.65%	0.22%
91	Cell culture	26482719	65.90%	6.98%	27.12%	0.14%
